# Medicine effectiveness comparisons of different physicotherapeutics for stroke patients with dysphagia: A protocol for systematic review and network meta-analysis

**DOI:** 10.1097/MD.0000000000022183

**Published:** 2020-09-18

**Authors:** Ying Li, Runmin Li, Jia Xiao, Yan Hu, Rongrong Xie, Yanyan Yuan, Dongying Li

**Affiliations:** a School of nursing, Nanchang University, Jiangxi; b College of Traditional Chinese Medicine, Shandong University of Traditional Chinese Medicine, Jinan; c Intensive Care Unit, The Second Affiliated Hospital of Nanchang University, Nanchang, Jiangxi, People's Republic of China.

**Keywords:** dysphagia, network meta-analysis, physical therapies, protocol, stroke

## Abstract

**Background:**

Oropharyngeal dysphagia is a common disorder after stroke. Physical therapy has been widely used in the rehabilitation of patients with dysphagia after stroke. Due to the lack of randomized trials directly comparing the efficacy of various physical therapies directly, the relative efficacy of these methods is difficult to determined. Therefore, we intend to conduct a network meta-analysis to evaluate the benefits of these physical therapies.

**Methods:**

According to the retrieval strategies, randomized controlled trials (RCTs) on physical therapies for stroke patients with dysphagia will be obtained from CNKI, Wan Fang Data, PubMed, Web of science, Embase databases and Cochrane Library, regardless of publication date or language. Studies were screened based on inclusion and exclusion criteria, and the Cochrane risk bias assessment tool will be used to evaluate the quality of the literature. The network meta-analysis will be performed in Markov Chain Monte Carlo (MCMC) method and carried out with Stata14 and OpenBUGS14 software. Ultimately, the evidentiary grade for the results will be evaluated.

**Results:**

This study will compare the efficacy of physical therapies in the treatment of stroke patients with dysphagia and suggests a reasonable clinical choice.

**Conclusion:**

Our findings will provide references for future guidance developing and clinical decision.

## 1 Introduction

Stroke is a serious global public health problem, with one in four people affected over their lifetime, and is the second leading cause of death and third leading cause of disability in adults worldwide.^[1,2]^ Stroke is defined as a neurological deficit attributed to acute focal injury of the CNS by vascular causes, including hemorrhagic stroke and ischemic stroke.^[3,4]^

Dysphagia is a common disorder after stroke, with an incidence of 37% to 78%.^[2,5,6]^ Most poststroke patients can recover swallowing function with routine therapies; however, for approximately 11% to 50% of patients, inability to swallow is a long-term disability.^[7,8]^ Chronic dysphagia may lead to several complications, such as dehydration, malnutrition and aspiration pneumonia, which consequently result in poor quality of life.^[9]^ Dysphagia results in a threefold increased risk of aspiration pneumonia and a mortality rate that is 5.4 times higher than that of patients without dysphagia.^[10]^ It is very important for dysphagia to be treated with drugs and physical therapy. At present, increasing attention has been paid to physical therapy. Physical therapy is effective, noninvasive, easy to perform, and low cost and has few side effects.^[10]^ The currently used physical treatment methods for dysphagia include posture training, swallowing exercises, neuromuscular electrical stimulation, and noninvasive brain stimulation.

Randomized controlled trials and meta-analyses have been reported frequently in dysphagia. However, due to the limitations of scale and research design, it is difficult to directly rank the efficacy of different physical therapies. As a branch of traditional meta-analysis, network meta-analysis integrates the existing research and forms an evidence network that can indirectly compare the therapeutic benefits.^[2,11,12]^ This study will use network meta-analysis to evaluate the effectiveness and safety of different physical therapy methods for stroke patients with dysphagia, and its conclusion will further guide clinical practice and strive for the best interests of patients.

## 2 Methods

### 2.1 Objectives and registration

This systematic review will aim to evaluate the effect and safety of different physical therapy methods for stroke patients with dysphagia. Our protocol has been registered on the International Platform of Registered Systematic Review and Meta-Analysis Protocols (INPLASY). The registration number was INPLASY202070125 (DOI:10.37766/inplasy2020.7.0125).

### 2.2 Ethics and communication plan

Our article is a secondary study, which does not involve the recruitment of patients, data collection, and ethical considerations. We will publish the results of network meta-analysis in the form of journal papers or conference papers.

### 2.3 Qualification criteria

According to the principle of PICOS, the inclusion and exclusion criteria of literature were determined.

#### 2.3.1 Types of participants

According to the diagnostic criteria of stroke (1996) revised by the fourth national Cerebrovascular Disease Conference of Chinese Medical Association or the Chinese stroke rehabilitation guidelines, dysphagia was diagnosed by CT or MRI.^[13,14]^

#### 2.3.2 Types of interventions and controls

The control group was given routine nursing, routine drug treatment and routine rehabilitation. On the basis of the control group, the experimental group added physical therapies such as electrical stimulation, cold stimulation and biofeedback. Due to the heterogeneity of acupuncture and moxibustion treatment, various types of acupuncture treatment were excluded.

#### 2.3.3 Types of outcomes

The primary outcomes should include the National Institutes of Water Swallow Test (Japan), Swallowing function assessment (SSA), the number of effective patients (the objective criteria for cerebral), and the number of adverse reactions. Secondary outcomes will include the Activity of Daily Living Scale, Health Stroke Scale (NIHSS) score.

#### 2.3.4 Types of studies

The included studies will be RCTs in this systematic review regardless of publication status and language. Animal trials, systematic review, case reports and studies with incorrect designs or incomplete data will be excluded.

### 2.4 Data sources and retrieval strategy

Studies will be obtained from the China National Knowledge Infrastructure (CNKI), Wan Fang Data, Chinese Scientific Journals Database (VIP), PubMed, CBM, Embase, Web of science and Cochrane Library, regardless of publication date or language. The databases will be retrieved by combining the subject words with random words. Taking PubMed as an example, the retrieval strategy is shown in Table 1. The search terms will be adapted appropriately to conform to the different syntax rules of the different databases.

**Table 1:**
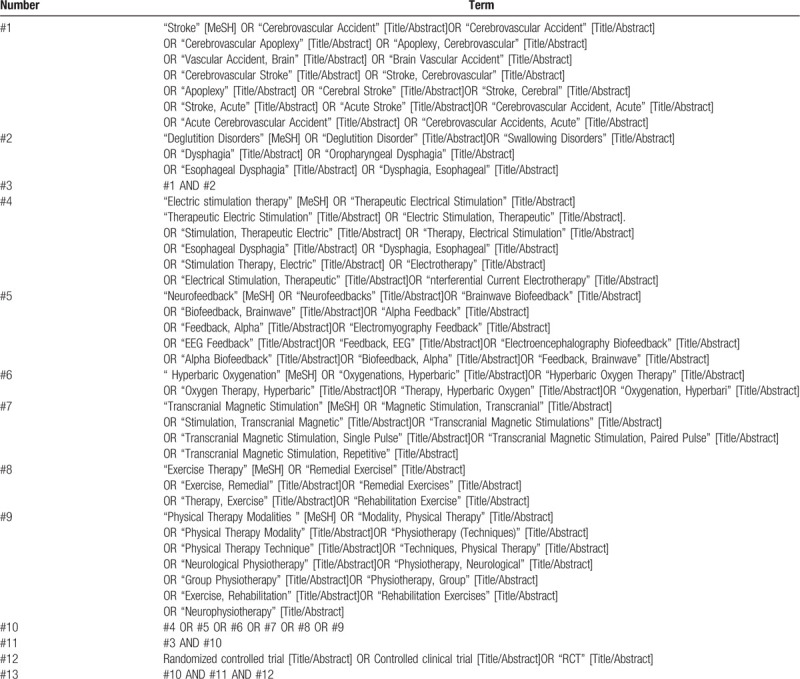
Retrieval strategy of PubMed.

### 2.5 Study selection and data extraction

**Figure 1:**
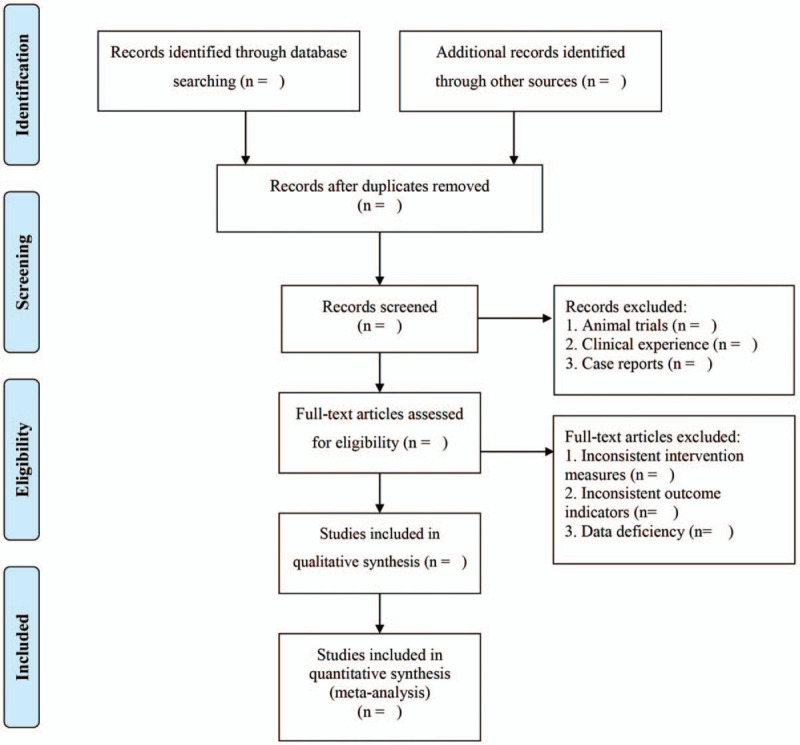
PRISMA flow chart.

EndNoteX9 will be used to manage the retrieved studies. As shown in Figure 1, the study selection will be divided into two steps and completed by two researchers (Jia Xiao and Rongrong Xie). Preliminary screening: eliminate repeated and unqualified studies by reading the title and abstract. Rescreening: read through the full text and select the studies according to the inclusion and exclusion criteria. According to the Cochrane Handbook for Systematic Reviews of Interventions, the two researchers (Yan Hu and Yanyan Yuan) will extract the author, publication time, participant number, age, race, Intervention measures in control group, Intervention measures in experimental group, course of treatment and outcome indicators, fill in the data extraction table, and Compare the baseline levels of patients.

### 2.6 Risk of bias assessment

Two researchers (Yan Hu and Jia Xiao) assessed the quality of the included RCTs independently by utilizing the Cochrane risk of bias assessment tool. As specified by Cochrane Handbook V.5.1.0, the following sources of bias were considered: random sequence generation, allocation concealment, participant blinding, intervention blinding, outcome assessor blinding, incomplete outcome data, selective reporting, and other sources of bias. Each domain was rated as having a high-risk, low-risk or unclear-risk of bias as appropriate.^[15]^ The two reviewers resolved any differences through discussion. If no consensus can be reached, consult experts in the field and refer to their opinions.

### 2.7 Statistical analysis

#### 2.7.1 Traditional meta-analysis

Direct comparisons of CHI efficacy will be performed using Review Manager 5.3. The outcomes will be mainly represented by the mean difference (MD) or odds ratio (OR) with 95% confidence intervals. For continuous data, the pooled standarized mean differences(SMDs) and their corresponding 95% confidence intervals (95%CIs) were used to assess the strength *P* < .05 was considered as statistically significant. The Cochrane *Q*-test and *I*^2^ statistics were used to assess heterogeneity. When *P* < .1 or *I*^2^ > 50%, which indicates statistical heterogeneity, a random-effects model will be used to calculate the outcomes; otherwise, a fixed-effects model will be considered.

#### 2.7.2 Network meta-analysis

A network evidence diagram will be drawn to visually represent the comparisons between the studies. The size of the nodes represents the number of participants, and the thickness of the edges represents the number of comparisons. Stata14 and OpenBUGS14 Software will be used to carry out Bayesian network meta-analysis. Bayesian inference will carried out using the Markov chain Monte Carlo (MCMC) method, the posterior probability will be inferred from the prior probability, and estimation and inference will be assumed when MCMC reaches a stable convergence state. As a result, the rank of different physical therapy methods effect will be presented by the surface under the cumulative ranking curve (SUCRA).

The node splitting method is used to evaluate the inconsistency between direct comparison and indirect comparison.^[12]^ The choices between consistent and inconsistent models and between fixed-and random-effect models will be made by comparing the deviance information criteria (DIC) for each model.^[16,17]^

#### 2.7.3 Subgroup and sensitivity analysis

If there is high heterogeneity in the included studies, we will perform subgroup analyses to explore the differences in age, sex, race, lesion location, and course of the Intervention time. To ensure robustness of the combined results, sensitivity analyses will be performed to assess the impact of studies with a high risk of bias. We will compare the results to determine whether lower-quality studies should be excluded.

#### 2.7.4 Publication biases

We will use funnel plots to identify whether there will be small study bias if 10 or more studies are included. Asymmetry in the funnel plot will suggest the possibility of small study effects, and the results of analysis will be explained cautiously.

### 2.8 Quality of evidence

The Grading of Recommendations, Assessment, Development and Evaluation (GRADE) approach will be used in evaluating evidence quality. Considerations of evidence quality assessment include study limitation, consistency of effect, imprecision, indirectness, and publication bias. The evidence quality will be classified into 4 levels (high, medium, low, and very low).^[18]^

## 3 Discussion

Physical rehabilitation training is very important for the rehabilitation of stroke patients with dysphagia. At present, physical rehabilitation training has been widely used in clinical research. However, the selection of clinical rehabilitation should be based on strict experimental design and objective evaluation and on the level of evidence.

## Author contributions

**Conceptualization:** Ying Li, Runmin Li, Dongying Li.

**Data curation:** Jia Xiao, Rongrong Xie, Yanyan Yuan.

**Formal analysis:** Ying Li, Runmin Li.

**Methodology:** Dongying Li.

**Software:** Jia Xiao, Rongrong Xie.

**Supervision:** Dongying Li.

**Writing – original draft:** Ying Li, Runmin Li.

**Writing – review & editing:** Dongying Li.

## Abbreviations

Abbreviations: CI = confidence interval, CNKI = China National Knowledge Infrastructure, Development and Evaluation, DIC = deviance information criteria, GRADE = the Grading of Recommendations, Assessment, MCMC = Markov Chain Monte Carlo, MD = mean difference, NIHSS = National Institutes of Health Stroke Scale, OR = odds ratio, RCTs = clinical randomized controlled trials, SD = standard deviation, SUCRA = surface under the cumulative ranking curve.
